# The Ontario Climate Data Portal, a user-friendly portal of Ontario-specific climate projections

**DOI:** 10.1038/s41597-020-0489-4

**Published:** 2020-05-19

**Authors:** Huaiping Zhu, Jinliang Liu, Xiaolan Zhou, Xiaoyu Chen, Xin Qiu, Richard L. Bello, Ziwang Deng

**Affiliations:** 1 Laboratory of Mathematical Parallel Systems (LAMPS), Department of Mathematics and Statistics, York University, Toronto, ON M3J 1P3 Canada; 2 Department of Earth and Space Science and Engineering, York University, Toronto, ON M3J 1P3 Canada; 3 SLR Consulting (Canada) Ltd, 150 Research Lane, Suite 105, Guelph, ON N1G 4T2 Canada; 4 Department of Geography, York University, Toronto, ON M3J 1P3 Canada

**Keywords:** Environmental health, Projection and prediction

## Abstract

An easily accessible climate data portal, http://yorku.ca/ocdp, was developed and officially launched in 2018 to disseminate a super ensemble of high-resolution regional climate change projections for the province of Ontario, Canada. The spatial resolution is ~10 km × ~10 km and temporal resolution is one day, UTC. The data covers 120 years from 1981 to 2100. This user-friendly portal provides users with thousands of static and interactive maps, decadal variation trend lines, summary tables, reports and terabytes of bias-corrected downscaled data. The data portal was generated with an emphasis on interactive visualization of climate change information for researchers and the public to understand to what extent climate could change locally under different emission scenarios in the future. This paper presents an introduction to the portal structure and functions, the large extent of the datasets available and the data development methodology.

## Introduction

The impacts of climate change are global in scope and unprecedented in scale. Governments and enterprises are making plans to mitigate and adapt to the changing climate. It has become critically important for researchers and policy makers to have easy access to climate change information at the right spatial and temporal scales for their studies and practices. Many users do not have the necessary expertise to collate the information into a form for their needs^1^. Since 2013, we have developed several versions of the data portal to disseminate Ontario-specific climate change data. We have been updating the portal with our newest results and improving the presentation and content based on feedback from users. In general, larger ensembles generate more robust conclusions. The conclusions based on a small ensemble of climate projections usually contains uncertainties that cannot be ignored and are prone to various potential errors caused by imperfections in models and downscaling methods^2^. Therefore, in addition to our data, some other limited data sources available for climate change projections for the Province of Ontario, Canada, are also used to develop the latest version of the data portal. Considering climate data/projections are one of the critical inputs for any climate change related risk/vulnerability assessment, the development of the user-friendly Ontario Climate Data Portal (OCDP) with the robust Ontario-specific data would better support practitioners’ climate change related risk/vulnerability assessments.

An effective web data portal for climate change will significantly facilitate the dissemination of information. For example, web data visualization provides a powerful tool for presenting climate change information, which makes abstract climate change information easier to understand; a web-enabled data warehouse also provides faster and easier access for different users. Currently, the web has been widely used to disseminate climate change information. Examples include the World Climate Research Program’s Coupled Model Intercomparison Project Phase 5 (CMIP5)^3^, the National Oceanic and Atmospheric Administration (NOAA)^4^, the National Center for Environmental Prediction (NCEP)^5^, the European Centre for Medium-Range Weather Forecasts (ECWMF)^6^, the Pacific Climate Impacts Consortium (PCIC) Archive Downscaled GCMS Portal^7^, the Coordinated Regional Climate Downscaling Experiment (CORDEX) data portal^8^, the Environment and Climate Change Canada (ECCC) data portal^9^ and the Climate Change Data Portal (CCDP^10^). This paper describes the latest version of our data portal, the OCDP.

In this study, we use the IPCC endorsed multiple model/scenario approach to address uncertainties in future projections. We developed a super ensemble (collectively 209 members) and high resolution (~10 km × ~10 km) regional climate projections for Ontario based on all available and creditable data sources at the time this study occurred; the source data for the supper ensemble covers the entire Province of Ontario and are generated by credible developers using methods published in peer-reviewed journals. These projections were developed based on the IPCC Fifth Assessment Report (AR5) data under all four Representative Concentration Pathways (RCPs), namely RCP 2.6, RCP 4.5, RCP 6.0 and RCP 8.5^11^; and this super ensemble serves as a common set of the most current and relevant projections. It will be used in future climate change related research and practices, and therefore will greatly improve the consistency and comparability among climate risk assessments for Ontario. To further improve data communication and access, the OCDP is developed with intuitive visualization for the general public and policy makers, and extensive data (over 10 TB) downloadable for climate academia and practitioners. Because the sizes of data linked to most web pages are large, the primary target users are desktop users. OCDP differs from other available portals in the following aspects: (1) it disseminates a set of super ensemble projections which permits addressing the uncertainty in assessments; (2) projections are based on a super ensemble (209 members) which combines members generated by both statistical and dynamical downscaling; (3) the dynamical downscaling components (42 members from 7 RCMs) in our projections are expected to better account for the impacts from local geophysical features (such as the Great Lakes, and Niagara Escarpment), which are critical to Ontario’s local climate.

## Results

**Fig. 1:**
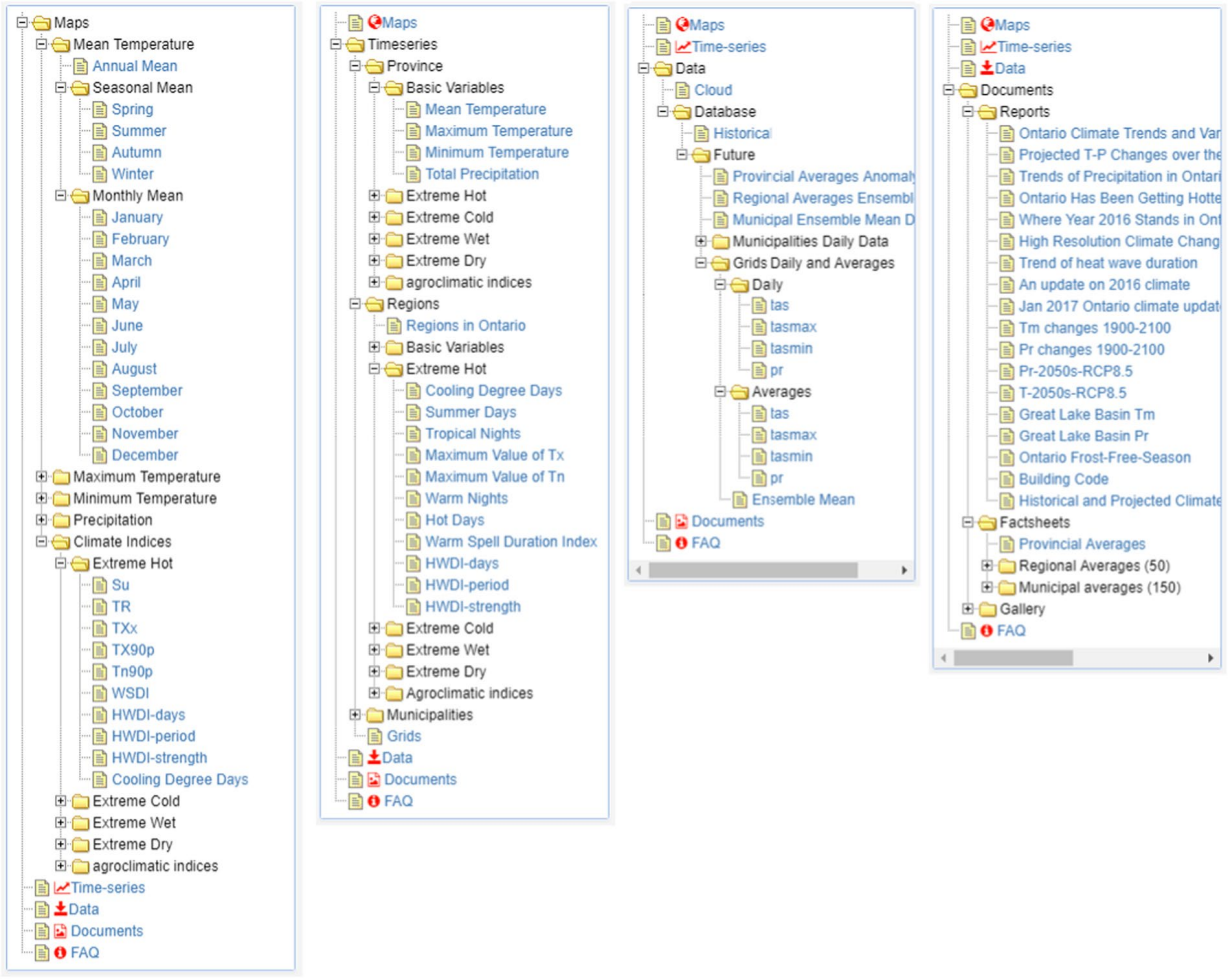
Overview of the OCDP. The expandable tree menus list all available climate change maps, time series, downloadable data sets and special reports.

OCDP contains five major components: *Introduction, Maps*, *Time series*, *Data* and *Documents* that cover different types of climate change information for Ontario. The *Introduction* page provides brief descriptions of methodologies and data sources used for generating the super ensemble of Ontario-specific climate change projections. We next describe the remaining four components of OCDP: *Maps*, *Time series*, *Data* and *Documents* (Fig. 1).

### Maps

The Maps component includes both static and interactive climate maps over Ontario (left column of Fig. 1). These maps are based on summarized data over the Province (represented by 8964 grid points at ~10 km resolution). The maps for the four basic variables (daily mean, minimum and maximum temperature, and daily total precipitation) are temporal averages or changes for three periods. Following common definitions, the three periods are the reference period (1986–2005) and two future periods for 2040-2069 (2050s: representing the mid-term of the 21^st^ century) and 2070–2099 (2080s: representing the last term of the 21^st^ century)^12–15^. The values for the two future periods are changes relative to the absolute value for the reference period. The statistics of changes are based on the sub ensemble of each of the four RCPs respectively; the maps for 38 climate indices are plotted and presented in the same manner as for the four basic variables. The colour of the maps follows the standard colour scheme commonly used in climate science community, i.e., blue (brown) represents low temperature (precipitation) and red (green) represents high temperature (precipitation). For easy comparison, we use unified colour scales among all the plots of the same type, although on some occasions the unified colour scheme may dim the contrast among neighbouring grids.

**Fig. 2:**
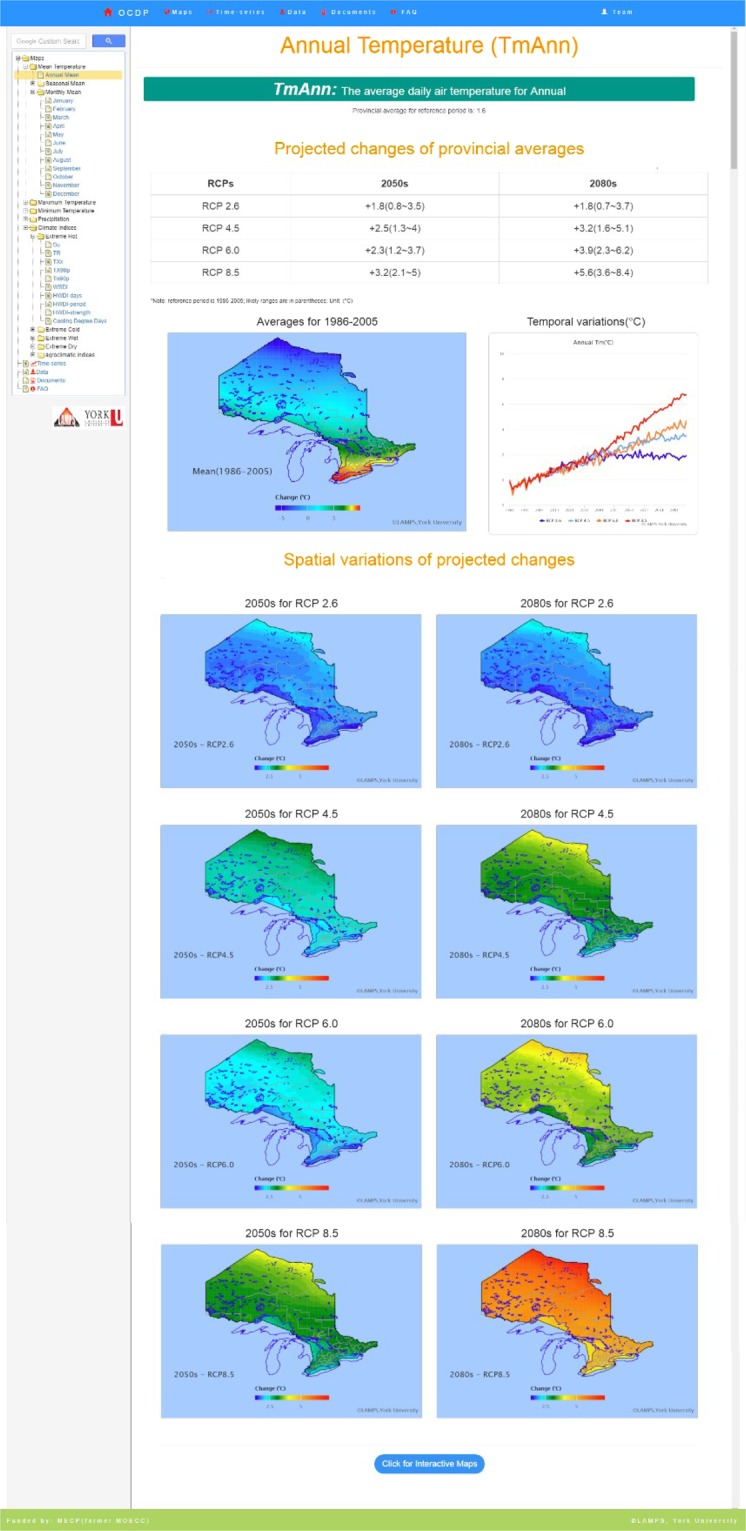
A screenshot of Maps component.

Each page for a variable/index consists of four subsections with its definition at the top followed by a brief summary table for the Province as a whole, then the static maps and a clickable link to access the interactive maps (Fig. 2). The summary (top section of Fig. 2) provides the Provincial averages of the climate variables (averages/indices) and their corresponding confidence intervals for the three periods, to address the uncertainty. In addition, we also provide a map of the variable on the left for the reference period and a time series on the right to show temporal variation of the variable for a period of 120 years (1980–2100). Following the summary for the whole province, we present a section consisting of eight static maps to show changes of the variables for the two future periods relative to the reference period under the four RCPs. The same colour scheme is used in all of these change maps, so that users can easily compare the changes of the variable among periods and RCPs. When users click the button for Interactive Maps at the bottom of the page, the third sub-section will appear which shows nine interactive maps for users who are interested in the detailed information at each of the 8964 grid points over the entire province. These interactive maps allow users to click at any grid point on the maps to obtain detailed information at that grid point. When the mouse hovers over a grid point in the first map - Present Climate and a Summary of Projected Changes at each Grid, a popup table appears in the lower-left corner of the window that shows similar climate change statistics as in the Provincial summary table in subsection 1 but with a focus for that specific grid point. Users can download the maps as .png, .jpeg, .pdf or .svg format pictures or as tables in .csv, .xls or .txt format. This functionality is implemented by several free software such as highchart.js, highmap.js and export.js^16^. Since the data volume for the interactive maps is large, a clickable button is provided as an option at the bottom of the page (Fig. 2) for those who would like to access more detailed information; this has significantly improved the UX of the OCDP.

### Time series

Variations in climatic variables over decadal-to-century time scales are the most important indicator of climate change. A sparkline (a tiny chart) is suitable to show trends. In this project, we calculate decadal (10-year) averages of climatic variables at 10-year intervals (e.g., 1981–1990, 1991–2000, etc.) and present them as sparklines for each of the sub-regions and municipalities over Ontario. Due to the large volume of data, we only present ensemble means of these variables for each of the RCPs in this section.

### Data for downloading

Many scientists and climate change adaption policy makers need more detailed information and data to carry out additional analyses on their own. To meet this increasing demand, we provide the portal with more than 10 terabytes (TB) of data, including daily, monthly, annual and long-term averages of minimum, maximum and average temperatures and precipitation from each model under all RCPs; annual values of extreme climate indices are provided as well. All of these data are downscaled and bias-corrected so that users can directly make use of the data in their analyses and practices (e.g. climate change impact assessment for specific sectors). We also provide simple codes in popular program languages such as Python, Matlab and R for reading the data available from OCDP. The units for temperature and temperature change are in °C, precipitation are in mm, and for precipitation change maps are in %, unless a specific description is provided.

### Documents

In the Documents section, we present a description for each section of OCDP, a large number of summary tables for Ontario and some special reports. The description clarifies how to use the OCDP. The summary tables include a table for Ontario averages, 50 tables for census divisions and 151 tables for municipalities. Each table provides changes of the four basic variables and the 38 climate indices for the 2050s and 2080s under the 4 RCPs. Values in the tables for Provincial and regional averages are spatial averages over the grids within Ontario and each region, respectively. Values in the tables for municipalities are changes at the corresponding weather stations. In addition, we have created many specific reports to answer some common questions from the public and government agencies, and some examples of application of data from the portal. Examples include: “What are the projected temperature and precipitation over the Great Lakes Basin?”; “What will be the trend in frost free days in the future?”; and “How will climate change impact future building code updates?”, etc.

### Query tools and usage statistics

As shown in Fig. 1, the collapsible tree menu provides an easy way for users to reach different pages/data on the OCDP. Another query tool is a free version of the Google custom search engine on the OCDP. We input a web address and 3-5 key words for each page into the engine database. Thus, the customized engine can quickly find our pages on the OCDP. Google analytics is a valuable tool for monitoring web site usage. We have been using this tool to monitor usage of OCDP. OCDP has been under development since 2016 and officially published online on June 1, 2018. Since then, we have had 9,051 unique visitors access the site about 51,269 times. We have been constantly improving the portal based on feedback from visitors.

## Discussion

OCDP provides rich Ontario-specific climate change information; it has helped and will continue help policy makers, researchers and the public to better understand the local climate impacts in Ontario under global warming. Meanwhile it will become a valuable open repository of high resolution regional and local climate change data for academic users and other climate change practitioners. For example, Environmental Commissioner of Ontario^17^ has referred to many results in a report. Some local governmental agencies such as the Haliburton, Kawartha, Pine Ridge District Heath Unit^18^ and the Simcoe Muskoka District Health Unit^19^ have used the OCDP data for their health risk and vulnerability assessments; and City of Orangeville^20^ has used the OCDP projections to guide its climate change policy and adaptation plan development.

The ready-to-use summary tables for policy makers at provincial, sub-region and municipal levels, will be very helpful for users with limited resources or capacity to derive climate change information from raw data. The maps and time series figures will help users to understand spatial and temporal distribution of climate change. The targeted users of this portal are primarily practitioners and academic researchers who most commonly access OCDP from desktop computers; therefore, we have designed web pages to be as compact as possible. To provide rich information on each page, generally the interactive pages are linked to one or several large datasets, which will be automatically loaded to the user’s device and displayed by the user’s browser. However, this type of design has some display limitations for mobile phone users when loading the interactive maps, as it is slow, making the interactive table screen untidy. Therefore, we have blocked interactive maps to mobile users at present. We plan to improve the OCDP in future to make it more platform-friendly for smart phone users. Some future improvements for mobile device users include, but are not limited to, (1) providing an option for these users to turn on or off the interactive content on their devices; and (2) making the display less screen-size dependent.

Recently, ECMWF published a new generation reanalysis ERA5^21^ to replace the ERA-Interim reanalysis, which stopped being produced on 31 August 2019. The strength of ERA5 over ERA-Interim has been demonstrated in some comparison studies^22,23^. It is therefore necessary to update ERA-Interim with the ERA5 in the next version of the OCDP. It is expected that the AR6 will be published in 2022 and the CMIP6 data will be available shortly thereafter when we will begin updating the portal with this new information.

The data portal is designed to disseminate Ontario specific climate change data and targeted users include climate impact researchers, government policy makers and other stakeholders who are interested in climate change while having some underlying knowledge about climate systems. Consequently, the design of the data portal follows other professional data portals focusing on climate change information presentation. We have endeavored to prepare and present data using standard formats to facilitate further analysis. We anticipate that some of the products from the portal may still be difficult to understand for users who do not have basic climate change knowledge, but we strive to continue improving the portal to expand its use to as wide a range of users as possible. Some examples include, describing SI energy equivalents using indicators which are more straightforward, like water level change, growth in ice thickness or change in durations of periods like snow cover. At present, the portal focuses on future climate change projections, but some users are interested in historical climate change so we are planning to include historical analyses of climate change information as well.

## Methods

### Data sources

The super ensemble of climate projections was developed using multiple credible sources of data, including conventional weather station observations, comprehensive reanalysis, and statistical and dynamical downscaled data. Brief descriptions of each of these data sources are provided in the following sub-sections.

### Observations at conventional weather stations in ontario

Daily observational data from 151 conventional weather stations (http://lamps.math.yorku.ca/OntarioClimate/assets/Locations/station151.html) within Ontario were downloaded for the 25-year period (1981–2005) from the ECCC website (http://climate.weather.gc.ca/historical_data/search_historic_data_e.html). In Ontario, there are only 127 stations that have at least 15-year complete precipitation data (0 day with missing data) and 101 stations that have at least 15-year complete temperature data. In this study, following the World Meteorology Organization (WMO) “3 and 5 rule” (https://climate.weather.gc.ca/glossary_e.html), we consider a year as having complete data if both the total number of days with missing precipitation and the total number of days with missing temperature are less than or equal to 36 (i.e. 3/month × 12 month) in that year; consequently there are 151 stations meet this requirement. These observations are used to validate the modelled data and correct biases for each of the 151 municipalities. The reason for extending data from the 20-year standard reference period (1986–2005) to 25-years is boost the robustness of bias correction because there are usually not enough complete data in the 20-year for effective use in bias correction. The 15-year criterion sounds short but is still comply with WMO rules (https://climate.weather.gc.ca/glossary_e.html), and is the most appropriate for Ontario based on data availability.

### Comprehensive reanalysis data

The critical information needed in our downscaling model development is comprehensive reanalysis data. Two major advantages of comprehensive reanalysis data are that (1) it is of high resolution, and (2) it accounts for multiple sources of meteorological/climatological data (as opposed to only observations at conventional weather stations) via assimilation algorithms^24–26^; these advantages are more profound in vast regions like Northern Ontario, which has a very sparse network of conventional weather stations. There are currently multiple sources for high resolution daily reanalysis datasets that cover the entire province, including the ECMWF reanalysis climate data^24^, the NCEP North America Regional Reanalysis (NARR)^25^ and the NCEP Climate Forecast System Reanalysis (CFSR)^26^. The ECMWF ReAnalysis (ERA) data, ERA-interim, is a widely used reanalysis production, which provides surface data in 11 different resolutions. In this study, we used the 0.125° grid reanalysis data which is the highest spatial resolution provided by the ECMWF web applications server.

Previous studies show that this dataset fits observational data very well in Ontario and is suitable for Ontario climate change studies^27,28^. The daily data in this study is calculated based on 6-hourly average temperature and 12-hourly precipitation, and minimum and maximum temperature from ERA-interim reanalysis data.

### Statistically downscaled datasets

Majority members (162) of our super ensemble projections (209-member) are generated with statistical downscaling methods. These statistically downscaled members include those from the Pacific Climate Impacts Consortium (PCIC, https://pacificclimate.org/analysis-tools/pcic-climate-explorer) and from the Laboratory of Mathematical Parallel Systems (LAMPS) of York University. The data from PCIC (66 members) are generated with the Bias-Correction Spatial Disaggregation (BCSD)^29^ and Bias Correction/Constructed Analogues with Quantile mapping reordering (BCCAQ)^30^. The PCIC data covers only three of the four IPCC RCPs: RCP2.6, RCP4.5 and RCP8.5. The LAMPS daily temperatures and precipitation are downscaled from GCMs to the ERA-Interim grid points (0.125° *Lon* × 0.125° *Lat*) using a combination of localized ensemble optimal interpolation (EnOI) and bias correction^28^. The LAMPS data cover all four IPCC RCPs (96 members).

### Dynamically downscaled datasets

To better account for the impacts from local geophysical features (i.e. the Great Lakes and the Niagara Escarpment) on Ontario’s climate, our super ensemble includes 47 dynamically downscaled projections; since the cost of dynamical downscaling is expensive, research institutes often only run several models under one or two scenarios; these dynamically downscaled members are provided by the North America node of the Coordinated Regional Downscaling Experiment (NA-CORDEX, 22 members)^31^, University of Toronto (UofT, 16 members)^32^ and the University of Regina (CCDP, 9 members)^33^. The NA-CORDEX data archive contains outputs from regional climate model (RCM) runs over a domain covering most of North America using boundary conditions from global climate model (GCM) simulations in the CMIP5 archive. These simulations run from 1950-2100 with a spatial resolution of 0.22° (~25 km) or 0.44° (~50 km). Temperature and precipitation at daily and longer time scales are available^31,34^. The UofT members cover the entire Province of Ontario and the Great Lakes Basin, generated using the US Weather Research and Forecasting (WRF) model driven by different GCM simulations in the CMIP5 archive. UofT has results for the RCP8.5 scenario^32^ only. The data from the University of Regina are generated using the Regional Climate Model system (RegCM) and another model system PRECIS (Providing Regional Climates for Impacts Studies) at a resolution of 25 km under RCP4.5 and RCP8.5 emissions scenarios, driven by boundary conditions from CMIP5 archive^33^. These studies show their methods are valid in downscaling temperature and precipitation for Ontario^28–33^.

**Fig. 3:**
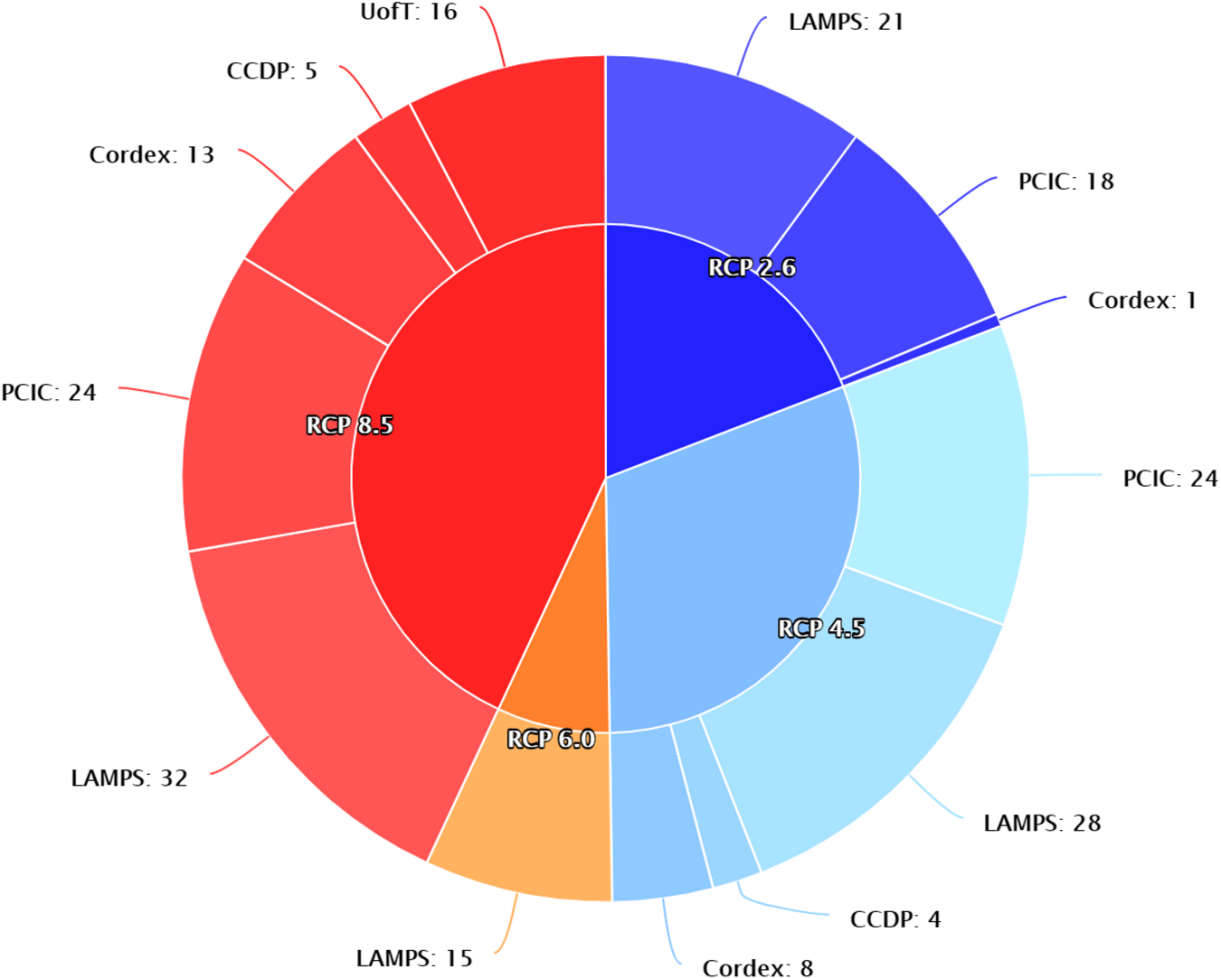
Composite of the super ensemble of high-resolution regional climate projections for Ontario.

**Fig. 4:**
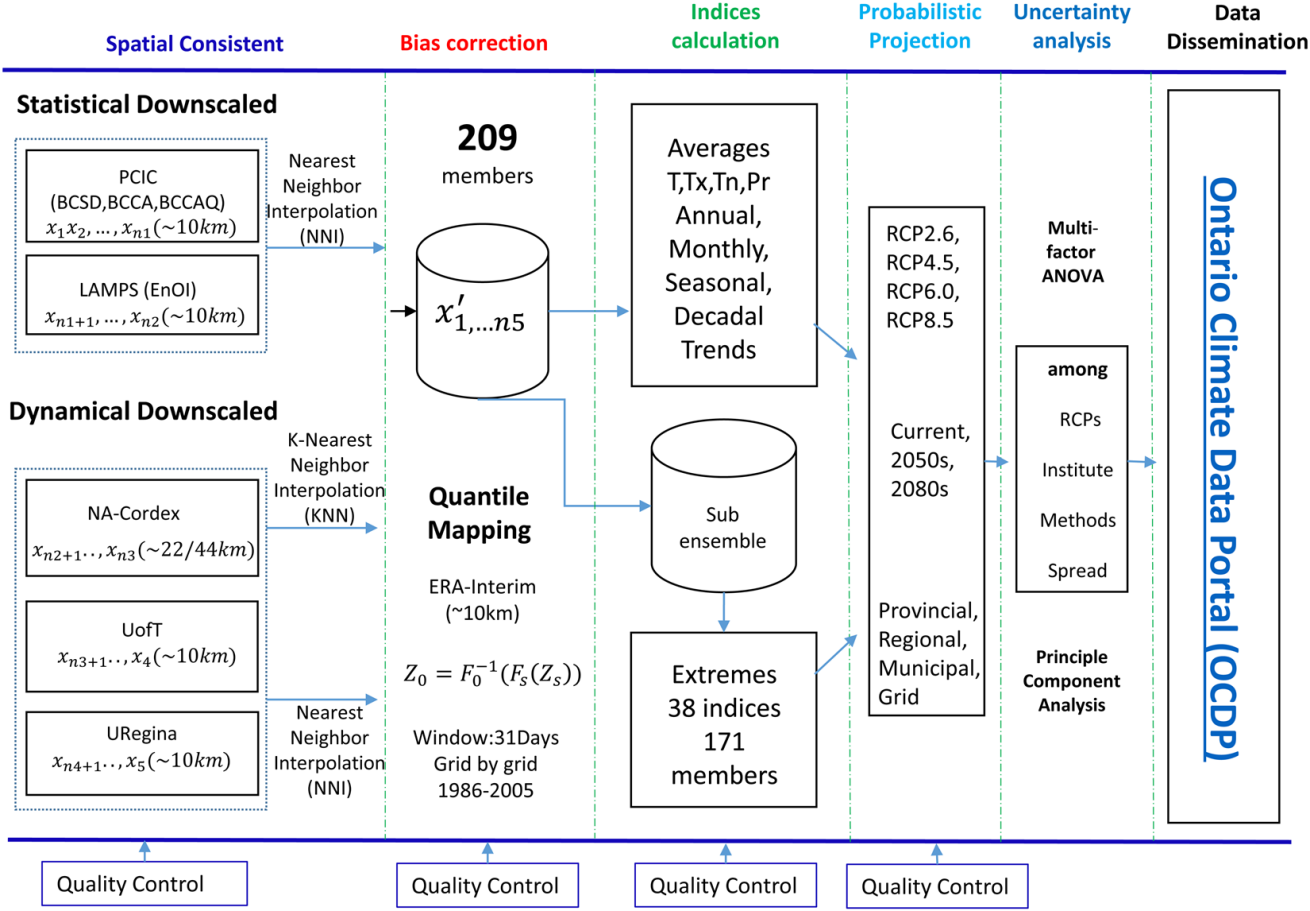
Flowchart illustrating the procedure of super ensemble development.

Ultimately, our super ensemble is developed using the 209 members from the five credible academic institutes, including 40(19.1%), 64(30.6%), 15(7.2%) and 90(43.1%) members for RCP 2.6, 4.5, 6.0 and 8.5, respectively (Fig. 3). This is by no means a simple combining procedure. To facilitate comparison and analysis of the ensemble data, all downscaled data are re-gridded to the common high-resolution (0.125°) ERA-Interim grid points and bias-corrected with the Quantile-Quantile mapping (QQ-mapping) method^35,36^. QQ-mapping equates cumulative functions of observed data and downscaled data in the reference period. Then biases are corrected in downscaled data under the assumption that this relationship applies to the future downscaled data. For grid data, we assume the reanalysis variables represent observations, and correct the bias grid by grid, variable by variable. For station data, we use available observations within the 25-year window (1981–2005) and corresponding downscaled data within the 25-year window to construct the relationship, and then correct biases in downscaled variables with this relation. The downscaled station data are interpolated from grid data by K-Nearest-Neighbors (KNN) algorithm^37^. A strict quality control is carried out to guarantee integrity of the data (Fig. 4), which will be described in the following sub-section.

### Data re-gridding and bias correction

In general, the following bias correction methods are used to adjust downscaled results: (1) linear transformation, (2) local intensity scaling (LOCI), (3) power transformation, (4) variance scaling, (5) distribution mapping and (6) the delta-change approach. In order to reduce model biases in frequency and amplitude, we apply different bias correction methods to different downscaled variables, i.e. temperatures (Tm, Tx and Tn) and precipitation data. For long term (monthly or longer time scale) averages, the linear scaling method (LS)^38–40^ and LOCI^41^ are used. These simple methods aim to perfectly match the average monthly mean of corrected values with that of observed ones within the reference periods. We then employ this relationship to correct downscaled future data. For daily data, we use the QQ-mapping method to adjust the amplitude and frequency^35,36^. Since QQ-mapping equates cumulative functions of observed data and downscaled data in the reference period, it has the benefit of accounting for biases in all statistical moments of downscaled temperature and precipitation^42^.

We chose the ERA-Interim ~10 km (0.125°) resolution grid network as the common grid network for the projections because more than 85% of our data are close to the grid points (i.e., LAMPS data are on the grid points and PCIC and UofT data are very close to the grid points). We used the simple K-Nearest-Neighbors (KNN) algorithm (K = 4 for this study)^37^ to re-grid all of the data to the common grid network and then carry out bias correction using the QQ-mapping method^35,36^. After bias correction, the total wet days and the total wet day precipitation of downscaled data exactly equals that in ERA-Interim data for the historical period (1986–2005). For daily extreme temperatures (minimum and maximum), we correct biases in the differences between the maximum/minimum and the average, then add the corrected differences to the corrected averages to get the full values. This method can avoid unrealistic situations in which minimum (maximum) temperature is higher (lower) than average temperature due to using the QQ-mapping directly to minimum (maximum) temperature. When downscaled values in the future are beyond the range of downscaled data for the reference period, a constant transfer function beyond the highest observed quantile was assumed^43,44^. The difference between the future downscaled value and the maximum value for the reference period is directly added to the highest observed value for the reference period.

### Indices calculations

To characterize climate at a location, variables describing averages of weather, as well as the indices describing other aspects of weather patterns such as anomalous, rare and extreme events are essential^45,46^. These climate indices allow a statistical study of variations of the dependent climatological variables, including analysis and comparison of time series, means, extremes and trends. Therefore, annual, seasonal and monthly averages and 38 climate indices based on the four basic variables from each simulation are carried out at each of the 8964 grid points. The climate indices include the commonly used 27-core climate indices defined by the Expert Team (ET) on Climate Change Detection and Indices (ETCCDI)^45,46^ and 11 additional indices defined by Canadian researchers. We provide a table for the definition of these indices (http://lamps.math.yorku.ca/OntarioClimate/index_app_data.htm#/indexDefinationTable). Downscaling models are generally evaluated with data for historical periods^47,48^. It is difficult to do effective validation on individual model results after bias correction based on the relationship between data utilized for the historical period. Fortunately, a large ensemble of multiple model results helps to reduce these concerns. The final product from this project, posted on OCDP, represents a common set of probabilistic projections of both long-term averages and extreme indices at a spatial resolution of ~10 km and various temporal scales (annual, seasonal, monthly and daily) for Ontario, Canada. Projections are developed for all four IPCC RCP emission scenarios (RCP 8.5, 6.0, 4.5, and 2.6). Most practitioners, including policy makers, would like climate change information specific to their own regions or communities. For these users we have prepared spatially averaged data. This includes, averages over the entire Province of Ontario, for each of the 50 census regions (http://lamps.math.yorku.ca/OntarioClimate/assets/Locations/locationMap_bk.html), for each of the 151 municipalities, and at each of the 8964 grid points across the province, which are available in corresponding sections on OCDP.

The values in the tables posted in the Document/Factsheet section on OCDP are determined following these steps: (1) calculate annual values for each ensemble member (model run); (2) calculate the temporal averages for 1990s, 2050s and 2080s, respectively; (3) average over the runs for each of the four RCPs and estimate the 50th percentile and the likely range (5–95th percentile range) of the values. For the 2050s and 2080s, only changes relative to the reference period are presented.

### Data portal design

We use several advanced web development tools to organize our data and make them available online in various formats, i.e., maps, pictures, tables or files. The software includes the basic web development languages such as hypertext markup language (HTML), cascading style sheets (CSS), and JavaScript. In addition, some professional software to implement specific functions, such as the highcharts.js and highmaps.js (https://www.highcharts.com/), are used for web data visualization; easyUI (https://www.jeasyui.com/index.php) is used for tree menu; W3.css (https://www.w3schools.com/w3css/) and Bootstraps (https://getbootstrap.com/) are used for improving user experience (UX) design; and Angular (https://angular.io/) is used for routing and navigation. All of these programs are open-source and free for academic use.

One picture is worth a thousand words. Data visualization is the presentation of data in a graphical format^49^. Spatial distribution of climate variables and indices can help users understand a local climate change signal but also the change relative to their neighbouring regions, because climate change is spatially correlated among neighbouring regions. Maps are the most effective tool for presenting the spatial distribution of climate change information. Therefore, in the OCDP, we currently provide about one thousand climate maps that offer ease-of-use for a wide range of users. The goal of the interactive maps is to show summarized climate change information at a specific grid point or location with tooltips as mouse-over-grid-point. Since there are many variables for different periods, it is difficult to show all the information on one tooltip. In this portion of the portal, we show an ensemble mean accompanied by the corresponding 5–95th percentile range of the variable over the three periods: the reference (Ref) period, 2050s and 2080s.

The base maps are generated using the geographic information system ArcGIS^49^. Since the portal is designed with client-side web development language, the data should be sent to the user’s computer to display; online data transmission speed is an important factor that affects UX. The major problem with interactive data portals driven by large volumes of data is data transmission from the portal server to the client’s browser. We have designed our portal to minimize data transmission at the user’s initial access to the portal.

In addition, since more than 85% of users of our earlier versions of OCDP were desktop users; in the updated data portal, higher priority is given to desktop users. Some functions of the portal are blocked for mobile users to avoid unexpected costs due to inadvertently large data requests. We have stored summary tables in the widely used comma-separated values (.csv) format, and large raw data in Matlab data (.mat) format with efficient file compression to reduce file sizes. For the convenience of users, we provide sample codes to read the downloaded data in three popular computer languages (Python, R and Matlab), found in the frequently asked questions (FAQ) section.

## Data Availability

The datasets of the projection ensemble have been deposited in public repositories. They can be found in Figshare^50^. The datasets include: (1) The base maps in.GeoJson format, (2) Ensemble mean of the four basic variables for 8964 grid points for 2050s and 2080s, (3) Ensemble mean of the climate extremes indices for 8964 grid points for 2050s and 2080s, (4) Ensemble mean of annual values averaged over the Province, (5) Ensemble mean of annual values averaged for each of the 50 sub-regions, (6) Ensemble mean of annual values for each of the 151 municipalities and (7) Decadal change of climate extremes indices for the 50 sub regions and 151 municipalities. In each sub dataset, there are many.csv files. See online documentation for more details about the dataset.

## Code availability

All codes associated with the data portal are open source; they are available on GitHub under the MIT license. We created the codes in two popular languages, JavaScript for the formal OCDP website and Python to facilitate data availability and interpretation. The GitHub repository hosts these codes. The 12 Python programs are designed for data reading, plotting, mapping and exporting on local server (https://github.com/LAMPSYORKU/OntarioClimateDataPortal/tree/master/pythonCode).

All of these Python files have also been uploaded to the Figshare data repository^50^. Users can directly clone and download the repository to their local machine and run the Python programs in the Jupyter notebook environment to manipulate the data. HTML5 and JavaScript files for the formal data portal can be found in Figshare^50^ and GitHub (https://github.com/LAMPSYORKU/OntarioClimateDataPortal/tree/master/JavaScripts). Users, who are interested in web application development, can download and use them for their own projects.

## References

[1] Weichselgartner J, Arheimer B (2019). Evolving Climate Services into Knowledge–Action Systems. Weather, Climate, and Society.

[2] Deser C, Phillips A, Bourdette V, Teng H (2012). Uncertainty in climate change projections: the role of internal variability. Climate Dynamics.

[3] Emori *et al*. CMIP5 data provided at the IPCC data distribution centre. Fact Sheet of the task group on data and scenario support for impact and climate analysis (TGICA) of the intergovernmental panel on climate change (IPCC), 8 (2016).

[4] Soreide *et al*. A climate data portal. In MTS/IEEE Oceans 2001. An Ocean Odyssey. Conference Proceedings (IEEE Cat. No. 01CH37295) (Vol. 4, pp. 2315-2317). IEEE (2001).

[5] Saha (2010). The NCEP climate forecast system reanalysis. Bulletin of the American Meteorological Society.

[6] Dee (2011). The ERA‐Interim reanalysis: Configuration and performance of the data assimilation system. Quarterly Journal of the royal meteorological society.

[7] Hamlet (2013). An overview of the Columbia Basin Climate Change Scenarios Project: Approach, methods, and summary of key results. Atmosphere-ocean.

[8] Giorgi F, Jones C, Asrar GR (2009). Addressing climate information needs at the regional level: the CORDEX framework. World Meteorological Organization (WMO) Bulletin.

[9] Mrozewski, T. Climate change data. Bulletin-Association of Canadian Map Libraries and Archives (ACMLA), (**162**), 20–24 (2019).

[10] Wang X, Huang G, Liu J, Li Z, Zhao S (2015). Ensemble projections of regional climatic changes over Ontario, Canada. Journal of Climate.

[11] Van (2011). The representative concentration pathways: an overview. Climatic change.

[12] Carter, T. R., M. Hulme & M. Lal. Guidelines on the use of scenario data for climate impact and adaptation assessment. 69 (1999).

[13] Moss RH (2010). The next generation of scenarios for climate change research and assessment. Nature.

[14] Vliet Van, Michelle TH, Wiberg David, Leduc Sylvain, Riahi. Keywan (2016). Power-generation system vulnerability and adaptation to changes in climate and water resources. Nature Climate Change.

[15] Giorgi Filippo (2016). Enhanced summer convective rainfall at Alpine high elevations in response to climate warming. Nature Geoscience.

[16] Shahid, B. Highcharts essentials. *Packt Publishing Ltd*. (2014).

[17] Dianne Saxe, Environmental Commissioner of Ontario, Annual Greenhouse Gas (GHG) Progress Report - Facing Climate Change http://docs.assets.eco.on.ca/reports/climate-change/2016/2016-Annual-GHG-Report-EN.pdf. (2016).

[18] Shikaze, S. Climate Change Health Vulnerability and Adaptation Assessment, (2019).

[19] Simcoe Muskoka District Health Unit. Assessing health impacts and vulnerabilities due to climate change within Simcoe Muskoka.(2019).

[20] Orangeville, O. Climate projections indicate that Orangeville is going to experience warming temperatures, increased variability in precipitation and more frequent extreme weather events.(2019).

[21] Copernicus Climate Change Service (C3S): ERA5: Fifth generation of ECMWF atmospheric reanalyses of the global climate. Copernicus Climate Change Service Climate Data Store (CDS), date of access, https://cds.climate.copernicus.eu/cdsapp#!/home (2017).

[22] Wang Caixin, Graham RobertM, Wang Keguang, Gerland Sebastian, Granskog MatsA (2019). Comparison of ERA5 and ERA-Interim near-surface air temperature, snowfall and precipitation over Arctic sea ice: effects on sea ice thermodynamics and evolution. The Cryosphere.

[23] Hoffmann L (2019). From ERA-Interim to ERA5: the considerable impact of ECMWF’s next-generation reanalysis on Lagrangian transport simulations. Atmospheric Chemistry and Physics.

[24] Dee (2011). The ERA‐Interim reanalysis: Configuration and performance of the data assimilation system. Quarterly Journal of the royal meteorological society.

[25] Mesinger (2006). North American regional reanalysis. Bulletin of the American Meteorological Society.

[26] Saha (2010). The NCEP climate forecast system reanalysis. Bulletin of the American Meteorological Society.

[27] Deng Z (2016). Trend in frequency of extreme precipitation events over Ontario from ensembles of multiple GCMs. Climate dynamics.

[28] Deng Z, Liu J, Qiu X, Zhou X, Zhu H (2018). Downscaling RCP8. 5 daily temperatures and precipitation in Ontario using localized ensemble optimal interpolation (EnOI) and bias correction. Climate dynamics.

[29] Wood AW, Leung LR, Sridhar V, Lettenmaier DP (2004). Hydrologic implications of dynamical and statistical approaches to downscaling climate model outputs. Climatic change.

[30] Werner AT, Cannon AJ (2016). Hydrologic extremes–an intercomparison of multiple gridded statistical downscaling methods. Hydrology and Earth System Sciences.

[31] Mearns *et al*. The NA-CORDEX dataset, version 1.0. NCAR Climate Data Gateway. *Boulder (CO): The North American CORDEX Program* (2017).

[32] Peltier WR, d’Orgeville M, Erler AR, Xie F (2018). Uncertainty in Future Summer Precipitation in the Laurentian Great Lakes Basin: Dynamical Downscaling and the Influence of Continental-Scale Processes on Regional Climate Change. Journal of Climate.

[33] Wang X, Huang G, Lin Q, Nie X, Liu J (2015). High‐resolution temperature and precipitation projections over Ontario, Canada: a coupled dynamical‐statistical approach. Quarterly Journal of the Royal Meteorological Society.

[34] Diaconescu, E. P., Mailhot, A., Brown, R., & Chaumont, D. Evaluation of CORDEX-Arctic daily precipitation and temperature-based climate indices over Canadian Arctic land areas. *Clim Dyn*., 10.1007/s00382-017-3736-4 (2017).

[35] Maraun D (2013). Bias correction, quantile mapping, and downscaling: Revisiting the inflation issue. Journal of Climate.

[36] Cannon AlexJ, Stephen RSobie, Trevor QMurdock (2015). Bias correction of GCM precipitation by quantile mapping: how well do methods preserve changes in quantiles and extremes?. Journal of Climate.

[37] Altman NS (1992). An introduction to kernel and nearest-neighbor nonparametric regression. The American Statistician.

[38] Teutschbein C, Seibert J (2012). Bias correction of regional climate model simulations for hydrological climate-change impact studies: Review and evaluation of different methods. Journal of Hydrology.

[39] Fang G, Yang J, Chen YN, Zammit C (2015). Comparing bias correction methods in downscaling meteorological variables for a hydrologic impact study in an arid area in China. Hydrology and Earth System Sciences.

[40] Terink W, Hurkmans RTWL, Torfs PJJF, Uijlenhoet R (2010). Evaluation of a bias correction method applied to downscaled precipitation and temperature reanalysis data for the Rhine basin. Hydrology and earth system sciences.

[41] Schmidli, J., Frei, C., Vidale, P. L. Downscaling from GCM precipitation: a benchmark for dynamical and statistical downscaling methods. *Int J Climatol* **26**, 679–689 PL (2006).

[42] Hayhoe (2008). Regional climate change projections for the Northeast USA. Mitigation and Adaptation Strategies for Global Change.

[43] Maraun D (2016). Bias correcting climate change simulations-a critical review. Current Climate Change Reports.

[44] Boé J, Terray L, Habets F, Martin E (2007). Statistical and dynamical downscaling of the Seine basin climate for hydro‐meteorological studies. International Journal of Climatology: A Journal of the Royal Meteorological Society.

[45] Karl TR, Nicholls N, Ghazi A (1999). CLIVAR/GCOS/WMO workshop on indices and indicators for climate extremes: Workshop summary. Climatic Change.

[46] Zhang X, Hegerl G, Zwiers FW, Kenyon J (2005). Avoiding inhomogeneity in percentile-based indices of temperature extremes. Journal of Climate.

[47] Maraun (2015). VALUE: A framework to validate downscaling approaches for climate change studies. Earth’s Future.

[48] Ayar (2016). Intercomparison of statistical and dynamical downscaling models under the EURO-and MED-CORDEX initiative framework: present climate evaluations. Climate dynamics.

[49] Schmidts, M. Esri ArcGIS Desktop Associate: Certification Study Guide: Compatible with ArcGIS 10.1 443 and ArcGIS 10.0. *Esri Press* (2013).

[50] Zhu H-P (2020). figshare.

